# A Computation Method Based on the Combination of Chlorophyll Fluorescence Parameters to Improve the Discrimination of Visually Similar Phenotypes Induced by Bacterial Virulence Factors

**DOI:** 10.3389/fpls.2020.00213

**Published:** 2020-02-26

**Authors:** Valérian Méline, Chrystelle Brin, Guillaume Lebreton, Lydie Ledroit, Daniel Sochard, Gilles Hunault, Tristan Boureau, Etienne Belin

**Affiliations:** ^1^Emersys, SFR 4207 QUASAV, IRHS, UMR1345, Université d'Angers, Angers, France; ^2^ImHorPhen, SFR 4207 QUASAV, IRHS, UMR1345, Université d'Angers, Angers, France; ^3^Phenotic Platform, SFR 4207 QUASAV, IRHS, UMR1345, Université d'Angers, Angers, France; ^4^Laboratoire HIFIH, UPRES EA 3859, SFR 4208, Université d'Angers, Angers, France; ^5^Laboratoire Angevin de Recherche en Ingénierie des Systèmes, Université d'Angers, Angers, France

**Keywords:** imaging analysis, chlorophyll fluorescence parameters, Bhattacharyya distance, hierarchical clustering, biotic stress

## Abstract

Phenotyping biotic stresses in plant-pathogen interactions studies is often hindered by phenotypes that can hardly be discriminated by visual assessment. Particularly, single gene mutants in virulence factors could lack visible phenotypes. Chlorophyll fluorescence (CF) imaging is a valuable tool to monitor plant-pathogen interactions. However, while numerous CF parameters can be measured, studies on plant-pathogen interactions often focus on a restricted number of parameters. It could result in limited abilities to discriminate visually similar phenotypes. In this study, we assess the ability of the combination of multiple CF parameters to improve the discrimination of such phenotypes. Such an approach could be of interest for screening and discriminating the impact of bacterial virulence factors without prior knowledge. A computation method was developed, based on the combination of multiple CF parameters, without any parameter selection. It involves histogram Bhattacharyya distance calculations and hierarchical clustering, with a normalization approach to take into account the inter-leaves and intra-phenotypes heterogeneities. To assess the efficiency of the method, two datasets were analyzed the same way. The first dataset featured single gene mutants of a *Xanthomonas* strain which differed only by their abilities to secrete bacterial virulence proteins. This dataset displayed expected phenotypes at 6 days post-inoculation and was used as ground truth dataset to setup the method. The efficiency of the computation method was demonstrated by the relevant discrimination of phenotypes at 3 days post-inoculation. A second dataset was composed of transient expression (agrotransformation) of Type 3 Effectors. This second dataset displayed phenotypes that cannot be discriminated by visual assessment and no prior knowledge can be made on the respective impact of each Type 3 Effectors on leaf tissues. Using the computation method resulted in clustering the leaf samples according to the Type 3 Effectors, thereby demonstrating an improvement of the discrimination of the visually similar phenotypes. The relevant discrimination of visually similar phenotypes induced by bacterial strains differing only by one virulence factor illustrated the importance of using a combination of CF parameters to monitor plant-pathogen interactions. It opens a perspective for the identification of specific signatures of biotic stresses.

## Introduction

In recent years, plantx phenotyping has been significantly evolving. High-throughput plant phenotyping platforms have been developed to answer to the rapid improvement of plant genomic technologies. Time consuming expert-based approaches of traditional phenotyping is moving toward a technology-based approaches providing automatic and quantitative measurements of biotic or abiotic stresses.

Imaging analysis applied to plant phenotyping is a component of this evolution. Measurements based on automatic image analysis could provide higher throughput, accuracy, and reproducibility than human visual inspections (Bock et al., 2008). Imaging analysis can be applied in various parts of plant phenotyping domain, such as the characterization of plant structure at a given instant, the quantification of plant growth over time or the monitoring of plants interactions with the environment or with pathogens. Plant structure and growth are now accessible with various 3D imaging techniques (Fang et al., 2009; Jahnke et al., 2009; Dhondt et al., 2010; Alenya et al., 2011; Zhu et al., 2011; Bellasio et al., 2012; Paproki et al., 2012), while imaging of plant health is accessible with various functional imaging techniques (see Li et al., 2014; Mahlein, 2016 for recent reviews). Thermal, near infrared reflectance, hyperspectral reflectance and chlorophyll fluorescence imaging (CF imaging) are among the most popular imaging techniques for monitoring plant health.

CF imaging is of special interest as it can be considered as a non-invasive and non-destructive method to efficiently phenotype the impact of biotic (Baron et al., 2012; Rousseau et al., 2013; De Torres Zabala et al., 2015; Zhou et al., 2015; Montero et al., 2016; Perez-Bueno et al., 2016; Pineda et al., 2018) or abiotic stresses (Honsdorf et al., 2014; Mishra et al., 2014; Bresson et al., 2015; Sebela et al., 2018) on the photosystem II of plants. Based on an active imaging technique with illumination flashes, sequences of many images are acquired and exploited to then evaluate CF parameters. These CF parameters are studied both for basic research on photosynthetic processes (Genty et al., 1989, 1990; Lichtenthaler et al., 2005; Baker, 2008), or alternatively for applied purposes, such as screening for phenotypes of resistance to abiotic and biotic stresses. Contrary to the study of abiotic stresses, only few CF parameters have been exploited when studying biotic stresses (Gorbe and Calatayud, 2012). Among all CF parameters available, *F*_*v*_/*F*_*m*_ and *NPQ* are commonly used for studying biotic stress (Baron et al., 2016; Kalaji et al., 2017). These parameters could give an efficient pre-symptomatic measure of the impact of several pathogen (Csefalvay et al., 2009; Pineda et al., 2011). However, contrasts obtained may differ among the numerous CF parameters used. Therefore, the use of only a subset of CF parameters may limit the ability to discriminate visually similar phenotypes (Berger et al., 2007; Pineda et al., 2008).

Bacteria belonging to the genus *Xanthomonas* are associated to plants, and numerous strains are responsible for disease on many important crops, such as rice, bean, soybean, tomato, sugarcane, wheat, oilseed rape, as well as on model plants, such as *Arabidopsis thaliana*. Even though more than 400 plant species may be infected by strains belonging to the genus *Xanthomonas*, a single strain only displays a narrow host range, restricted to one or several plant species. Even though most strains of *Xanthomonas* spp isolated from plants were described as pathogenic, non-pathogenic strains of *Xanthomonas* spp have been also been isolated and described, receiving an increasing attention in the recent years (Cesbron et al., 2015; Essakhi et al., 2015; Garita-Cambronero et al., 2016; Merda et al., 2016, 2017).

Among the multiple virulence factors that have been described for *Xanthomonas* spp strains, the type 3 secretion system (T3SS) encoded by the *hrp* gene cluster, is known to play a central role in the interactions with plants. Indeed, the inactivation of the T3SS usually abolishes the virulence of pathogenic *Xanthomonas* spp strains on their host plants. On the other hand, the acquisition of a T3SS by non-pathogenic bacteria may constitute an evolutionary step toward the emergence of novel plant pathogenic bacterial strains (Manulis and Barash, 2003). For example, strain 7698R of *Xanthomonas cannabis* is non-pathogenic on bean, its host of isolation. When inoculated on the non-host plant *Nicotiana benthamiana*, strain 7698R induces a rapid necrosis similar to the hypersensitive reaction (HR) observed in non-host resistance. The complementation of this non-pathogenic strain with a plasmid harboring genes encoding a T3SS, suppressed the onset of this HR-like necrosis, and only a mild chlorosis could be observed on inoculated tissues at 6 days post-inoculation (6 dpi) (Meline et al., 2019). Such a suppression of a defense layer could constitute a first step toward the emergence of novel pathogenic strains.

This secretion system enables the injection of numerous bacterial effector proteins called Type 3 effectors (T3Es) directly into the plant cells, that collectively suppress the plant defenses and subvert the plant's physiology to the benefit of the bacteria (Li et al., 2002; Büttner, 2016; Jacques et al., 2016; Wei and Collmer, 2018). Repertoires of T3Es vary importantly among strains of *Xanthomonas* belonging to a same species, and were reported to correlate to some extend with the host specificity of strains (Hajri et al., 2009, 2012). Knowledge on the functions and cellular targets in plant cells of some single T3Es has considerably increased over the last decade. Numerous targets in various compartments of the plant cell were described (for review Büttner, 2016). For example, once injected inside the plant cells, the T3E *XopAC* of *Xanthomonas campestris* uridylylates the cytoplasmic kinase BIK1, which blocks the transduction pathways leading to plant immunity and promote bacterial virulence on *Arabidopsis thaliana* (Feng et al., 2012; Guy et al., 2013a,b). Another example is that of TAL effectors of *Xanthomonas* spp, enter the plant nucleus, recognize and bind specific DNA sequences and induce the expression of plant genes (Boch et al., 2014). Plant immunity and more globally the physiology of plant cells may also be altered by T3Es targeting other cell organelles, such as cytoskeleton and stromules, or chloroplasts (Büttner, 2016; Erickson et al., 2018).

However, in most cases, functions of T3Es and their targets in plant cells still remain elusive. Importantly, mutants in single T3E genes often hardly display any phenotype visible to the eye, which may hinder deciphering their role in the interaction between plants and bacteria (Mutka and Bart, 2015). Therefore, innovative tools are needed to better phenotype the impact of single T3Es on plant tissues.

In the present paper, we propose an approach based on CF imaging aiming at the discrimination of the behavior of such mutants on leaves. To this purpose, we developed a computation method based on the combination of multiple images of CF parameters. This approach aims at using the information contained in all the images of CF parameters, without prior selection nor focusing on the physiological processes involved. As such, the approach developed in the present study is mainly intended to setup screening methods for phenotyping closely related biotic stresses on plants.

## Materials and Methods

### *Xanthomonas* Mutant Strains

Mutant strains of *Xanthomonas* were already described in Meline et al. (2019). Strain 7698R is an environmental strain belonging to the species *X. cannabis* isolated from bean seeds, but not pathogenic on bean. As largely documented in Meline et al. (2019), when inoculated on *N. benthamiana*, this strain induces a necrosis of leaf tissues at 6 dpi. Strain 7698R was complemented with the plasmid pIJ3225 that carries a 20 kb *hrp* cluster encoding a major bacterial virulence determinant: the Type 3 Secretion System and four Type 3 Secreted Proteins (Arlat et al., 1991). On *N. benthamiana* at 6 dpi, the complemented strain 7698R pIJ3225 does not induce any necrosis, the inoculated tissues only display a chlorotic phenotype. Derivatives of pIJ3225 harboring a Tn5 insertion that inactivates one gene in the *hrp* cluster were characterized in Meline et al. (2019). These derivatives were transformed into strain 7698R to obtain strains 7698R pIJ3225::Tn5 (G9, G2, F2, F15, C3, or G1). After inoculation on *N. benthamiana* leaves, Tn5 insertions G9 and G2 restore the onset of the necrosis, the Tn5 insertions F2, F15, and C3 partially restore the necrosis, whereas Tn5 insertion G1 does not alter the phenotype conferred by pIJ3225. Strain 7698R and complemented strains 7698R pIJ3225::Tn5 were used in the present paper for inoculation on *N. benthamiana* and subsequent CF imaging at 3 dpi. The complete list of Tn5 derivatives used is reported in Supplementary Table 1.

### Cloning of Bacterial Virulence Factors

Six T3E genes (*xopAF, xopL, xopG, xopV, xopT*, and *xopAK*) of the sequenced strain *Xanthomonas citri* pv. *fuscans* CFBP 4834 were chosen for cloning into Gateway vectors, for subsequent transient expression in *N. benthamiana*. These T3E genes were chosen as previous knowledge suggests they are involved in various biological processes (Darrasse et al., 2013). Hence they constituted a good set of candidates for setup of an approach aiming at discriminating the impact of various virulence factors on plants. The *xopG* and *xopT* genes were chosen because they often are found in highly aggressive strains of *X. citri* on bean. In the genome of the model strain *X. citri* pv. *fuscans* CFBP 4834, these genes are flanked by insertion sequences suggesting that they may be horizontally transferred to other bacteria (Rousseau et al., unpublished data). The *xopV* and *xopAK* genes were chosen as they were proposed by Moreira et al. (2010) to be involved in the specificity of symptoms caused by *X. citri*. The *xopL* gene was described to impact stromule formation in plant cells, and is widely distributed among sequenced model strains of *Xanthomonas*. Conversely, the *xopAF* gene is poorly distributed among the sequenced model strains of *Xanthomonas*, and its distribution suggests this gene may be involved in tissue specificity of *Xanthomonas* strains (Bogdanove et al., 2011). Sequences of these T3Es were amplified by PCR using adequate primers summarized in Supplementary Table 2, and *AccuPrime*^*TM*^ Taq DNA Polymerase High Fidelity. Amplified sequences were cloned in pENTR/D-TOPO vector using pENTR Directional TOPO cloning kit (Invitrogen). Then, cloned sequences were transferred to the pB7WG2 binary vector, obtained from VIB (Belgium) (Karimi et al., 2002) using Gateway LR clonase II (Invitrogen). The nucleotide sequences of all constructs were confirmed by sequencing.

### *Agrobacterium tumefaciens*-Mediated Transient Expressions

Strain EHA105 of *A. tumefaciens* was transformed with the binary vector pB7WG2 containing the bacterial genes of interest fused to *gfp*. The subsequent derivatives of strain EHA105 were used to prepare an inoculum (see subsection Preparation of Inocula and Inoculation procedures) infiltrated into fully expanded leaves to perform transient expressions of the cloned genes in *N. benthamiana*. The transient expression of six T3Es and β-glucuronidase (GUS) were performed. To control the efficiency of the transient expression process, we revealed the GUS activity of samples of leaf tissues inoculated with the EHA105 pB7FWG2-gus at 24 h post-inoculation (hpi), 36 and 48 hpi (data not shown). The GUS activity of samples was revealed using a buffer containing X-Gluc (1 mM), K_3_Fe(CN)_6_ (4 mM), K_4_[Fe(CN)_6_]·3H_2_O (0.05 mM), EDTA (10 mM), Na_2_HPO_4_ (50 mM) and NaH_2_PO_4_ (50 mM) in a buffer phosphate. The efficiency of the transient expression of each T3E gene was controlled by observation at 24 and 48 hpi of a GFP signal resulting from the expression of the *gfp* fusion obtained by the cloning the T3E gene of interest into pB7FWG2.

### Plant Material

*Nicotiana benthamiana* plants were grown and inoculated in environmentally controlled growth room under a 16 h photoperiod and 8 h dark period at 22°C and 80% of relative humidity under a light intensity of 100 μE·m^−2^·s^−1^ throughout the whole experiment. At the optimal developmental stage (6 weeks old) the plants had at least five fully developed true leaves.

### Preparation of Inocula and Inoculation

*Xanthomonas* mutant strains were cultured on classical media TSA (Trypticase Soy Agar: Tryptone 17 g·L^−1^; peptone soja 3 g·L^−1^; glucose 2.5 g·L^−1^; NaCl 5 g·L^−1^; KH_2_PO_4_ 5 g·L^−1^; agar 15 g·L^−1^; pH 7.2, supplemented with the adequate antibiotics) and incubated at 28°C. For the inoculation, bacterial suspensions were calibrated at 10^8^ cfu.ml^−1^.

*A. tumefaciens* was cultured into 20 mL of Luria Bertani medium (Tryptone 10 g·L^−1^; NaCl 10 g·L^−1^; Yeast extract 5 g·L^−1^) in the presence of selective antibiotics (50 mg L^−1^ spectinomycin and 50 mg·L^−1^ gentamycin), and grown overnight in a rotary shaker at 150 rpm and 28°C. Cells were harvested by centrifugation and resuspended to a final concentration of 0.3 OD600 in a solution containing 10 mM MgCl_2_, 10 mM MES, 10 g·L^−1^ sucrose, pH 5.6 and 150 μM acetosyringone and incubated at 28°C for 3 h before agroinfiltration.

For *Xanthomonas* or *Agrobacterium* inoculation, three fully expanded leaves per plant were inoculated by pressing the blunt end of a 1 ml needleless syringe to the lower side of the leaf while supporting the leaf with a gloved finger.

### Datasets

In a validation approach, two datasets were used and processed the same way to setup and assess the computation method.

The first dataset was used as ground truth dataset to validate the computation method. It featured 85 leaves of *N. benthamiana* at 3 dpi. Each leaf was inoculated with three controls and one tested-strain as described in Meline et al. (2019). The three controls were, (i) strain 7698R used as necrosis control, (ii) strain 7698R pIJ3225 used as chlorosis control, and (iii) mock-inoculated control used as no symptom control. The tested-strains were strains 7698R pIJ3225::Tn5 (G9, G2, F2, F15, C3, or G1). Each inoculated area was cropped and considered as an independent sample. Thereby, 340 samples (85 × 4) were generated. For each sample, 70 images of CF parameters were obtained. The total size of this first dataset was of 23800 images. As expected, Tn5 insertions G9 and G2 restored the onset of the necrosis, the Tn5 insertions F2, F15, and C3 partially restored the necrosis, whereas Tn5 insertion G1 led to chlorosis phenotype at 6 dpi. However, at 3 dpi, all the controls and tested-strains displayed phenotypes that could hardly be discriminated by visual assessment.

The second dataset was used to assess the relevance of the computation method to discriminate leaf tissues impacted by biotic stresses without any prior knowledge. It featured 108 leaves of *N. benthamiana* each inoculated with three controls and one tested-strain. The three controls were, (i) *A. tumefaciens* strain EHA105 not expressing any exogenous protein, (ii) transient expression of β-glucuronidase as non-deleterious exogenous protein control, and (iii) mock-inoculated control used as no symptom control. The tested-strains were *A. tumefaciens* EHA105 transiently expressing one of the *X. citri* pv. *fuscans* virulence genes (T3Es): *xopL*: L, *xopT*: T, *xopV*: V, *xopAK*: AK, *xopAF*: AF, *xopG*: G. Each inoculated area was cropped and considered as an independent sample. Thereby, 432 samples (108 × 4) were generated. For each sample, 70 images of CF parameters were obtained. The total size of this second dataset was of 30240 images. This second dataset is of interest as phenotypes cannot be discriminated by visual assessment: no necrosis nor chlorosis can be observed either at 3 or 6 dpi. Therefore, no prior knowledge can be made on the respective impact of the transient expression of each T3Es on leaf tissues.

### Chlorophyll Fluorescence Imaging System

Acquisition of fluorescence images is performed with a PSI Open FluorCam FC 800-O. The system sensor is a CCD camera with a pixel resolution of 512 by 512 and a 12-bit dynamic. The system includes 4 LED panels divided in 2 pairs. One pair provides an orange actinic light with a wavelength around 618 nm, with an intensity up to 400 μmol·m^−2^·s^−1^. The other pair provides a saturating pulse in blue wavelength, typically 455 nm, with an intensity up to 3, 000 μmol·m^−2^·s^−1^. The acquisition protocol is a quenching analysis protocol (Kolber et al., 1998), producing a raw file containing 70 images of CF parameters. A schematic description of the quenching protocol is proposed in Figure S1. To measure the parameter F_0_, a modulated light of 0.1 μmol·m^−2^·s^−1^ is used. Then orange actinic light with intensities of 20% of the 400 μmol·m^−2^·s^−1^ is used during the light-adapted period of 60 s. The protocol also provides 6 pulses of 0.8 s duration of blue saturating light with an intensity of 50% of the 3,000 μmol·m^−2^·s^−1^: 5 pulses during the light-adapted period and 1 pulse during the dark-relaxation period. The 50% of the saturating light pulse was considered as a good intensity as it provides a ratio (*F*_*m*_−*F*_0_)/*F*_*m*_ of 0.82 measured on non-inoculated area, and being closed to optimal value of 0.83 (Bjorkman and Demmig, 1987). This was measured after a dark adaptation of 30 min. The whole duration of the illumination protocol is about 95.8 s. For evaluating the performance of our computation method on datasets obtained with such protocol, the 70 images given in gray-level intensity are processed in batch to provide histograms of pixels that statistically represent regions of interest, i.e., the four areas of infection on the leaf. Distances between the histograms of each leaf are then calculated according to Bhattacharyya distance.

### Bhattacharyya Distance

The Bhattacharyya distance measures the similarity of two discrete probability distributions or histograms (Bhattacharyya, 1943). This measure, regularly used in classification problems in the field of computer vision (Kailath, 1943), determines the relative closeness of two histograms being considered. The Bhattacharrya distance is known to be particularly useful to give a contrast scalar directly connected to detection performance in noisy images (Goudail et al., 2004). The Bhattacharyya distance *B*_*d*_ is defined as:

$$B_{d}=-ln\sum \sqrt{\left(h_{A}h_{B}\right)}$$

where *h*_*A*_ and *h*_*B*_ are the normalized histograms for two different areas *A* and *B*.

### 3D Euclidean Distance

According to Deza and Deza (2009), the distance between two points (*p*,*q*) in a three-dimensional Euclidean space (*x, y, z*) is defined as

$$E_{d}=\sqrt{{\left(x_{p}-x_{q}\right)}^{2}+{\left(y_{p}-y_{q}\right)}^{2}+{\left(z_{p}-z_{q}\right)}^{2}}$$

In a three dimensional Euclidean space, we also defined intra-modality and inter-modality distances. A modality is composed of several images of the same kind of inoculation. For each modality, the intra-modality distance is the mean Euclidean distance between each image and the centroid of the modality, constituting the dispersion of the modality. The inter-modality distance is the Euclidean distance between the centroid of each modality. Such distances were useful to evaluate contribution of the addition of CF parameters for the clustering ability. The addition of parameters was performed using a sequential forward sequence method (SFS) (Agrawal and Srikant, 1995).

### Clustering Based on Dendrogram

We used hierarchical clustering algorithm based on a Ward linkage method (Ward, 1963). This method is the only one among the agglomerative clustering methods that is based on a classical sum of squares criterion, producing groups that minimize within group dispersion at each merging step (Murtagh and Legendre, 2014). In R, the Ward.D2 algorithm of the function hclust is the one attributed to Ward. This function requires Euclidean distances as input dissimilarities. Several studies point out that this method outperforms other hierarchical clustering methods (Blashfield, 1976; Hands and Everitt, 1987). In our case this method allows to cluster modalities with a better accuracy.

To evaluate the clustering abilities of dendrograms, we computed the sensitivity and the specificity of the clustering. According to Parikh et al. (2008), the sensitivity and the specificity can be expressed as:

$$\begin{array}{l} \text{sensitivity}=\frac{\text{true positive}}{\text{true positive + false negative}}\\ \text{specificity}=\frac{\text{true negative}}{\text{true negative + false positive}} \end{array}$$

where, for the sensitivity, a true positive represented an image of one modality (for instance necrosis control) classified in the correct corresponding cluster (necrosis phenotype cluster) and false negative represented an image of the same modality (necrosis control) classified in the other uncorrect clusters (chlorosis and mock-inoculated clusters). For the specificity, true negative represented an image of the two other modalities (chlorosis and mock-inoculated) classified in one of these two corresponding correct cluster (chlorosis phenotype and mock-inoculated phenotype cluster) and false positive represented an images of the two other modalities (chlorosis and mock-inoculated controls) classified in a uncorrect cluster (necrosis phenotype cluster).

## Results

### Distance Calculation Between Tested-Strain and Controls for Each Image of CF Parameter

**Figure 1:**
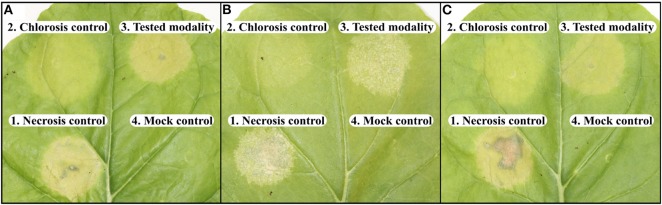
Visual observation of the inter-leaf heterogeneity at 3 dpi. **(A–C)** Were representative of the diversity of results obtained with the inoculations of the same strains on three different leaves. 1. necrosis: strain 7698R. 2. chlorosis: strain 7698R pIJ3225. 3. tested-strain: strain G9. 4. mock-inoculated.

In this study, phenotyping of interactions between *N. bethamiana* leaves and *Xanthomonas* strains was confronted to an inter-leaf heterogeneity. As illustrated in Figure 1, the intensity of the necrosis development, after inoculation with the same strain, could vary between leaves. To circumvent this inter-leaves heterogeneity, for both dataset, each tested modality was inoculated with their respective controls on each leaf as illustrated in Figure 1. In a normalization approach, each tested-strain was then characterized according to its distance with its respective controls on each leaf. This image processing procedure occurring in 4 steps was described for the computation of the first dataset there after.

**Figure 2:**
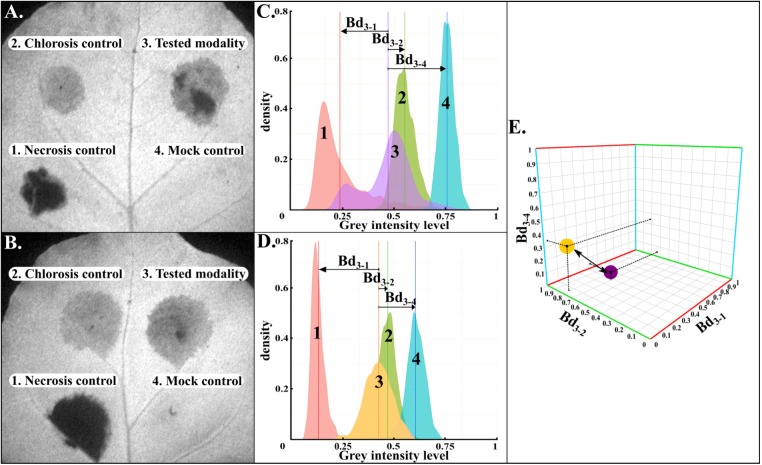
Method for the calculation of the distance between two different tested strains. **(A,B)** Illustrations of gray-level intensity images for two different leaves corresponding to *F*_*m*_ parameter. Gray-level intensity was coded between 0 and 1. The four areas corresponded to necrosis, chlorosis, tested strain, and mock-inoculated, respectively. **(C,D)** Extracted normalized histograms from areas 1 to 4. From these histograms, three Bhattacharyya distances (*Bd*_3−1_, *Bd*_3−2_, *Bd*_3−4_) were computed between tested strains and their three respective controls. Plant tissues could also display a heterogeneity intra the inoculated area. Bhattacharyya distances allowed to take into account each pixel value to consider this heterogeneity across each inoculated area. These three distances defined axes for a three-dimensional space where tested strains could be represented and compared to each others using Euclidean distance calculation. **(E)** Illustrated the three-dimensional representation and the three-dimensional Euclidean distance calculation between two tested strains (yellow and violet spots) for one image.

**Figure 3:**
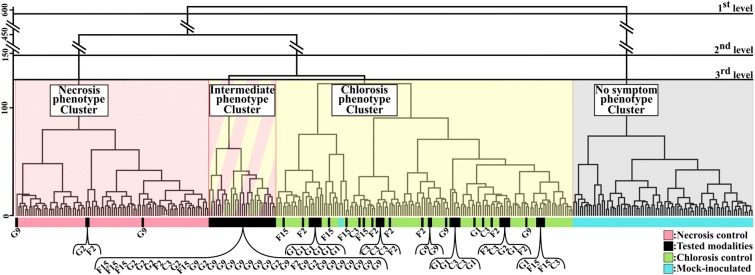
Clustering of samples of the first dataset according to the combination of the 70 images of CF parameters. Dendrogram based on three-dimensional Euclidean distance of 70 images combination and Ward agglomeration method. Black horizontal bars corresponded to the different levels of analysis of the dendrogram. Four clusters corresponding to the four phenotypes visually observed (necrosis, intermediate, chlorosis, and no symptom phenotypes) were obtained. Necrosis (pink) corresponds to leaf tissues inoculated with strain 7698R. Chlorosis (green) corresponds to leaf tissues inoculated with strain 7698R pIJ3225. Mock-inoculated (blue) corresponds to leaf tissues inoculated with water. Tested-strains (black) correspond to leaf tissues inoculated with strains G9, G2, F2, F15, C3, or G1.

Firstly, as illustrated in Figures 2A,B, from a gray-level intensity image, the normalized histograms of the cropped four areas were extracted (Figures 2C,D). Secondly, for each image of CF parameter, Bhattacharyya distances were calculated between the histogram of the tested-strain and each histogram of the three controls. As shown in Figure 2E, the three Bhattacharrya distances defined the axes of a three-dimensional plot, where each tested-strain modality can be represented. This representation had the advantage of taking into account the inter-leaves heterogeneity as each tested-strain was compared to its three controls on each leaf. Thirdly, our CF imaging protocol generated 70 images of CF parameters for each tested-strain. Therefore, each tested-strain can be characterized by an array of 70 three-dimensional Euclidean distances. Finally, tested-strains were then clustered according to their array of 70 three-dimensional Euclidean distances by hierarchical ascendant classification based on Ward's agglomeration method and represented with a dendrogram (Figure 3).

### The Discrimination of the Contrasted Phenotypes of Controls at 3 Days Post-inoculation Validated the Computation Method

To validate the computation method, the relevance of the 70 CF images combination method was evaluated through the discrimination efficiency of the three controls which induced contrasted phenotypes at 3 dpi. The first dataset was computed according to the method described in the previous paragraph. On the dendrogram of Figure 3, the three controls were clearly grouped in three distinct clusters referred to as necrosis phenotype, chlorosis phenotype and no symptom phenotype clusters. A first level of analysis revealed an efficient clustering of mock-inoculated control. Practically, all samples (except two samples) of this control were grouped into a single cluster (No symptom phenotype cluster). All the other clusters of the dendrogram are clearly discriminated from this latter cluster, which highlighted the impact of inoculation. At a second level of analysis, all samples of necrosis control and all samples of chlorosis control were respectively grouped into distinct clusters (Necrosis phenotype cluster and Chlorosis phenotype cluster, respectively). Results obtained using this method were in complete accordance with the observed phenotypes at 3 dpi, and with the expected phenotypes at 6 dpi for the controls used as ground truth (Meline et al., 2019).

To better assess the relevance of the approach, we compared the sensitivity and the specificity of the clustering using 70 images of CF parameters, or using a single parameter (Table 1). To perform clustering using single one, we chose *F*_*v*_/*F*_*m*_ or *NPQ*, as these parameters are commonly used in CF phenotyping (Baron et al., 2016; Kalaji et al., 2017). Related to *F*_*v*_/*F*_*m*_ and *NPQ*, two supplementary dendrograms were then built and shown in Figure S2. Using the combination of 70 images of parameters, the mock-inoculated control have been efficiently discriminated from other controls (sensitivity almost of 0.98 and specificity of 1). When using clustering based only on single parameter, contrasting results were obtained according to which parameter was used. Using the sole *F*_*v*_/*F*_*m*_ parameter, mock-inoculated control was also well-discriminated from other controls (sensitivity of 0.99 and a specificity of 1). Inversely, the sole *NPQ* parameter did not allow such an efficient discrimination as only 60% of the mock-inoculated control images were correctly classified into the no symptom phenotype cluster (sensitivity of 0.6). Moreover, by observing the associated dendrogram (dendrogram C. in Figure S2), we noted that the remaining 40% images of this control were grouped into a cluster where all the necrosis control images were also grouped. The necrosis and chlorosis controls have been efficiently discriminated using the combination of 70 images of CF parameters (sensitivities of 1 and specificities of 1 and 0.99, respectively). Using the sole *F*_*v*_/*F*_*m*_ parameter, the sensitivities of discrimination between necrosis and chlorosis phenotypes decreased down to 0.68 and 0.56, respectively, although its specificities remained high as 1 and 0.99, respectively. Such low sensitivities values could be explained with some mild necrosis or chlorosis phenotypes that were grouped in the intermediate phenotype cluster (dendrogram B. in Figure S2). Such results highlighted the interest of using a combination of 70 images of CF parameters to improve the discrimination of the three controls.

**Table 1:** Sensitivity (sens.) and specificity (spec.) of the clustering for the controls according to CF parameters for the two datasets.

**First dataset**						
	**Mock-inoc**.		**Chlorosis**		**Necrosis**	
**Parameter**	**Sens**.	**Spec**.	**Sens**.	**Spec**.	**Sens**.	**Spec**.
Combined 70	0.98	1	1	0.99	1	1
*F*_*v*_/*F*_*m*_	0.99	1	0.56	0.99	0.68	1
*NPQ*	0.60	1	0.75	1	1	0.57
**Second dataset**						
	**Mock-inoc**.		**GUS**		**EHA105**	
**Parameter**	**Sens**.	**Spec**.	**Sens**.	**Spec**.	**Sens**.	**Spec**.
Combined 70	1	0.87	1	0.65	0.87	0.67

*In the first dataset, controls are mock-inoculated, chlorosis and necrosis. In the second dataset, controls are mock-inoculated, GUS and EHA105*.

### Assessment of the Contribution of Each Image of CF Parameters in the Combination

**Figure 4:**
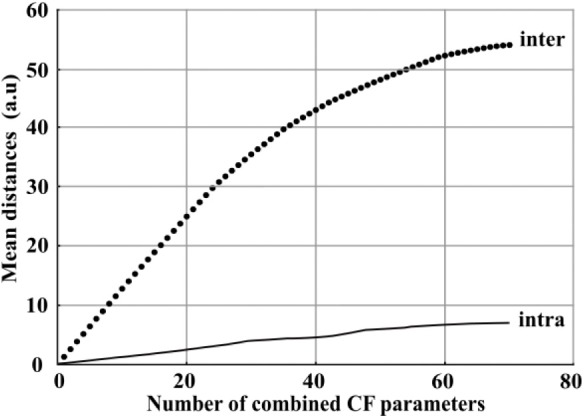
Evolution of the mean inter-modality distance (black spot) and the mean intra-modality distance (black line) between the three controls according to the number of images of CF parameters combined from 1 to 70.

According to the results exposed in last section, the combined 70 images of CF parameters provided a better clustering ability of the three controls than only one classical parameter, e.g., F_v_/F_m_ or NPQ in this study. To further confirm the relevance of the combination of images of CF parameters, it was therefore suitable to evaluate how the clustering ability is impacted according to the number of images of CF parameters which are combined. For these purpose, images were sequentially combined from 1 to 70 using sequential forward sequence method (SFS) based on the maximal mean inter-modality distance between the three controls. For each combination, we measured the evolution of the inter-modality and intra-modality distance between the three controls. For our application, the inter-modality distance can be considered as the useful information to discriminate the three controls and the intra modality distance represented the dispersion of all the images of one modality and can therefore be considered like degrading information as the dispersion increased. These mean distances are plotted in Figure 4.

The mean inter-modality distance increased quickly with the combination of images between 1 and 40. It then slowly reached a saturation level from 40 to 70. At the same time, the mean intra-modality distance remained at least five times lower than inter-modality distance values. As a consequence, while the combination of a large number of images increased the mean inter-modality distances, it was not a source of degrading information regarding the positive ratio between inter and intra-modality distance.

### Application of the Computation Method to Successfully Discriminate the Tested-Strains From the First Dataset

**Figure 5:**
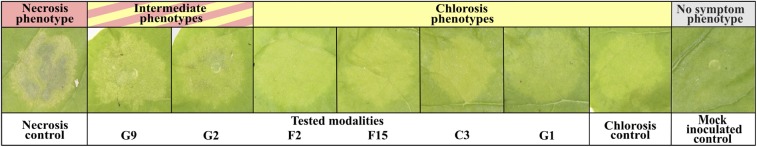
Visual observation of controls and tested-strains of the first data set at 3 dpi. Two phenotypes for the tested-strains (strains G9, G2, F2, F15, C3, G1) were identified as intermediate and chlorosis phenotypes.

At 3 dpi, among the six tested-strains of the first dataset, only two different phenotypes were visually annotated as shown in Figure 5. Tested-strains G9 and G2 were identified as intermediate phenotypes between necrosis and chlorosis phenotypes whereas the phenotypes of the other tested-strains (F2, F15, C3, G1) were identified as a chlorosis phenotype.

To assess the relevance of the computation method, we analyzed the clustering of these tested-strains (Figure 3). Samples from the tested-strains G9 and G2 were mainly grouped into a fourth cluster distinct from the three previously described clusters. This fourth cluster corresponded to samples displaying intermediate phenotypes. Such results were expected, as G9 and G2 corresponded to mutant strains for which Tn5 insertions fully inactivated the secretion system encoded on pIJ3225. In contrast Tn5 insertions of F2, F15, C3, G1 tested-strains only inactivated one secreted protein that transit through the secretion system encoded on pIJ3225. For the tested-strains (F2, F15, C3, G1), the inoculation resulted in phenotypes visually identified as chlorosis. Regarding these latter strains, the clustering ability of the method showed more differences than the visual observation. Tested-strains C3 and G1 were grouped almost exclusively into the chlorosis cluster. Whereas, F15 was grouped into intermediate and chlorosis clusters considering almost equal proportions, and F2 was mainly grouped into chlorosis cluster but with a small proportion also grouped in necrosis cluster. The clustering of these single gene mutants of Xanthomonas were in accordance with the expected impact of each inactivated gene in the ability of the plasmid pIJ3225 to suppress the necrosis development at 6 dpi, as described in Meline et al. (2019). Therefore, the combination of images of CF parameters allowed at 3 dpi a predictive discrimination between samples that could previously be discriminated only at 6 dpi. Proportions (in percentage) of samples of each tested-strain grouped into necrosis, chlorosis, intermediate or no symptom phenotype clusters were summarized in Table 2.

**Table 2:** Proportions of clustering of the tested strains for the two datasets, based on the combination of the 70 images of CF parameters.

**First dataset**				
**Tested strains**	**No symptom phenotype cluster (%)**	**Chlorosis phenotype cluster (%)**	**Intermediate phenotype cluster (%)**	**Necrosis phenotype cluster (%)**
G9	0	17	74	9
G2	0	11	78	11
F2	0	73	18	9
F15	0	60	40	0
C3	0	90	10	0
G1	0	100	0	0
**Second dataset**				
**Tested strains**	**Cluster 1 (%)**	**Cluster 2.1 (%)**	**Cluster 2.2.1 (%)**	**Cluster 2.2.2 (%)**
AF	0	11	39	50
L	0	0	11	89
G	6	11	44	39
V	11	11	17	61
T	0	6	0	94
AK	22	0	39	39

*Proportions are expressed in percentage. In the first dataset, tested strains are G9, G2, F2, F15, C3, G1. In the second dataset, tested strains are transient expression of xopAF: AF, xopL: L, xopG: G, xopV: V, xopT: T, xopAK: AK*.

### Application of the Computation Method on a Second Dataset to Discriminate Phenotypes for Which No Prior Knowledge Is Available

**Figure 6:**
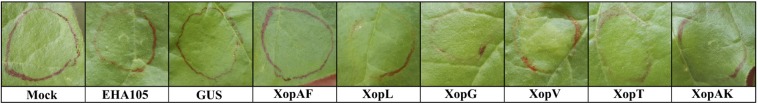
Visual observation of controls and tested-strains of the second dataset at 3 dpi. Mock-inoculated (Mock), *A. tumefaciens* strain no protein expressed (EHA105), the transient expression of β-glucuronidase protein (GUS) were considered as the three controls on each leaf. The six transient expression of T3E genes were considered as the tested-strains (transient expression of *xopAF*: AF, *xopL*: L, *xopG*: G, *xopV*: V, *xopT*: T, *xopAK*: AK, respectively). Constructs for transient expression are described in Supplementary Table 1.

**Figure 7:**
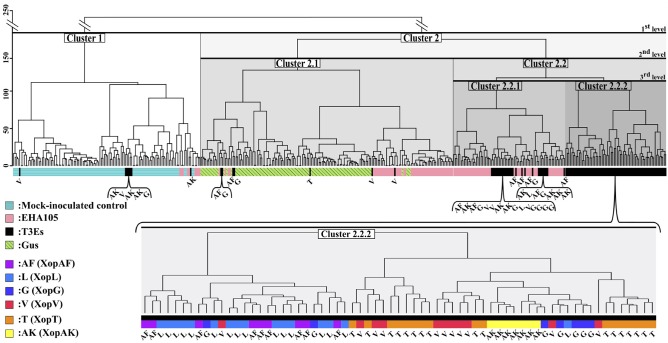
Clustering of samples of the second dataset according to the combination of the 70 images of CF parameters. Black horizontal bars corresponded to the different levels of analysis of the dendrogram. The lower part of the figure presents a focus on the cluster 2.2.2 regrouping the majority of the transient expression of T3Es considered as tested-strains.

To test the interest of the computation method on situations that cannot be discriminated by visual assessment and for which no prior knowledge is available, we used a second dataset composed of bacterial virulence factors (T3Es) transiently expressed on leaves (*xopAF*: AF, *xopL*: L, *xopG*: G, *xopV*: V, *xopT*: T or *xopAK*: AK). As shown in Figure 6, 3 dpi, no difference was observed visually between mock-inoculated control and the transient expressions of β-glucuronidase protein (GUS) or five T3Es among the six tested. Only transient expression of *xopV* could in rare case induce a mild chlorosis. The computation method was applied to the second dataset (Figure 7) and proportions (in percentage) of images of each tested-strain grouped into four clusters were summarized in Table 2.

At a first level of analysis, a relevant clustering of mock-inoculated control vs. leaf tissues inoculated with bacterial strains was obtained. All samples corresponding to mock-inoculated control were grouped in cluster 1 with a sensitivity equal to 1. Few samples corresponding to other modalities were misclustered, resulting in a specificity of 0.87 in cluster 1. Here again, plant tissues inoculated with bacterial strains could be discriminated from mock-inoculated strains in the absence of any phenotype visible to the eye (Figure 6). At a second level of analysis, the transient expressions of the GUS reporter protein could be discriminated from the transient expression of T3Es. Indeed, all samples of leaf tissues expressing GUS were clustered in cluster 2.1 whereas most samples of leaf tissues expressing T3Es were clustered in cluster 2.2. At this level, the method allowed to discriminate the impact on leaf tissues of presumably deleterious bacterial virulence proteins (T3Es) vs. non-deleterious exogenous proteins (GUS). The sensitivity of GUS clustering was of 1. Its specificity decreased down to 0.65 because of the presumably deleterious effect on leaf tissues of the inoculation with *A. tumefaciens* (EHA105). The sensitivity of EHA105 clustering in cluster 2.2 was of 0.87 with a specificity 0.67. The clustering ability in terms of sensitivity and specificity for these three controls (mock-inoculated, GUS, and EHA105) is summarized in Table 1. When further focusing, cluster 2.2.2 grouped 94, 88, and 61% images of leaf tissues inoculated with *xopT, xopL*, and *xopV*, respectively. Moreover, the computation method discriminated the transient expression of *xopL* vs. the transient expression of *xopT* and *xopV* (Figure 7, cluster 2.2.2).

## Discussion

CF imaging is a powerful technique to study quantitatively plant-pathogen interactions (Perez-Bueno et al., 2016; Pineda et al., 2018) and has been used to highlight potential physiological mechanisms underlying disease symptoms (De Torres Zabala et al., 2015; Zhou et al., 2015). However, some CF parameters are still very contentious (Kalaji et al., 2014) and caution must be exercised when attempting to interpret their significance (Baker, 2008; Murchie and Lawson, 2013). Moreover, careful setups of illumination protocols have to be considered for the assessment of physiological studies. In the context of our study, we developed a computational method to improve the discrimination between close phenotypes based on the combination of images of multiple CF parameters. In the present study, these multiple CF parameters are generated from non-optimized illumination protocols which preclude any physiological interpretation. However, this approach would be fully compatible with optimized illumination protocols. Such an optimization of illumination protocols would probably result in improved performances of the discrimination. The use of optimized illumination protocols in combination with the method described in the present paper may also allow the discrimination of distinct physiological status of plants. However, this latter is beyond the scope of this paper.

Although a large variety of CF parameters is available, only a subset of parameters have been used in the literature (Kalaji et al., 2014). Parameters empirically selected do not provide necessarily the best contrast between studied phenotypes. For instance, *F*_*v*_/*F*_*m*_ parameter is a relatively stable ratio as impacts of stress could be detected rather late (Lichtenthaler et al., 2005). *NPQ* parameter could be one of the most appropriate one to distinguish plant-pathogen interactions (Rodriguez-Moreno et al., 2008; Perez-Bueno et al., 2015). But it has also been shown to be an unstable temporal parameter (Bonfig et al., 2006; De Torres Zabala et al., 2015). Inversely to subjective selections of these parameters, the depicted computation method provided an objective method to exploit all the information from all the available images of CF parameters. However, although the present paper presents a computation method to gather all the information provided by multiple images of CF parameters, the illumination protocol remains to be optimized to provide more physiologically relevant information.

It had been considered that CF imaging and thermography techniques could have a lack of potential to identify specific diseases in contrary to RGB and hyperspectral imaging techniques (Martinelli et al., 2015; Mahlein, 2016). Indeed, taken individually, *F*_*v*_/*F*_*m*_ parameter could be impacted either by any abiotic or biotic stress factor like water deficit (Bresson et al., 2015) or bacterial disease development (Perez-Bueno et al., 2016). However, it has been shown that the inoculation of an avirulent *Pseudomonas syringae* pv. *glycinea* on soybean leaves was associated with a decrease in *F*_*v*_/*F*_*m*_ and an increase in *NPQ* parameters (Zou et al., 2005) whereas an inoculation of an avirulent *Pseudomonas syringae* pv. *tomato* on *arabidopsis thaliana* was associated with a decrease in both *F*_*v*_/*F*_*m*_ and *NPQ* parameters (Bonfig et al., 2006). Thereby, combination of several CF parameters could constitute a specific signature of visually similar stress, as reviewed in Baron et al. (2012).

During infections, plant-pathogen interactions could have effects on several mechanisms of the plant. Regulation of stomatal aperture could impact temperature dynamic which could be study using thermography imaging (Sankaran et al., 2013; Maes et al., 2014). Production of phenolic compounds involved in plant defense could be measured with blue green fluorescence (BGF) which can be study using Multi Color Fluorescence Imaging (MCFI) (Perez-Bueno et al., 2015; Montero et al., 2016; Ortiz-Bustos et al., 2017). CF imaging could measure performance of the photosystem II which could be altered by the development of a pathogen (Rousseau et al., 2015). Using several imaging techniques in parallel could improve the performance of detection and allowed pre-symptomatic identification of a stress. It could also be used to improve the discrimination abilities to identify disease signatures for a specific pathogen (Mahlein et al., 2012; Baron et al., 2016). The method presented in this study could be generically applied on a combination of multi-modal images.

Adding a temporal monitoring may improve the phenotyping for the characterization of the plant-pathogen interactions. Indeed, monitoring the temporal dynamic revealed useful to discriminate temporal spectral signatures of three foliar pathogens of barley leaves (Wahabzada et al., 2015). As well, Berger et al. (2007) showed that infections by virulent and avirulent strains of *Pseudomonas syringae* result in distinct temporal dynamics of CF parameters, although the same CF parameters were involved. For example, the response to *Pseudomonas syringae* pv. *tomato* DC3000 could be discriminated from the response to its mutant inactivated in the type 3 secretion system by a transient increase of *NPQ* parameter between 6 and 12 hpi (De Torres Zabala et al., 2015). Furthermore, spatio-temporal phenotyping of the response to virulent and avirulent strains of *Pseudomonas syringae* provided non-redundant information (Perez-Bueno et al., 2015). Therefore, combining our computation method with a temporal monitoring could improve significantly the discrimination of biotic stresses on leaves.

The aim of our paper is essentially to present and discuss a computation method combining for each sample 70 images of CF parameters, without any selection, and computing a normalization in order to take into account the inter-leaves and intra-phenotypes heterogeneities. We show its implementation and its usability to provide significant results, in the study-case of the discrimination of visually similar phenotypes induced by bacterial virulence factors. We showed our method to be useful for phenotyping of the impact of single T3E on plant tissues following transient expression. Any T3E could be tested this way and our approach could reveal of interest as well for phenotyping the impact on plant tissues of T3Es of other plant pathogenic bacteria, such as *Pseudomonas syringae* or *Ralstonia solanacearum*. The genericity of the present computation method also resides in the possibility to be applied systematically to any dataset where controls can be defined. In that way, controls can be defined within each leaf (as described in the present paper) or within each experiment if it is not possible to apply different stresses to the same leaf. Furthermore, the use of CF imaging for the phenotyping is widely documented for the study of both biotic (Chaerle et al., 2004) and abiotic stresses (Yusuf et al., 2010).

Although we showed in this study that the combination of a large number of CF parameters was not degrading information, we could not rule out that some parameters could carry redundant information. Moreover, it had been demonstrated that in some case, the combination of eleven or less CF parameters involved the most important fluorescence signatures and could be sufficient to classify tissues inoculated with different strains (Mishra et al., 2014; Cen et al., 2017). It could be interesting to process the datasets using selective and reductive methods (as decision tree for instance) to select and identify parameters which could discriminate tissues inoculated with different strains and to reduce inherent redundancy and overfeeding. However, we could consider that the present method remained an efficient alternative to such learning methods which could require large annotated datasets that can be tough to obtained in plant phenotyping domain. In that direction, further studies aiming at selecting the most informative CF parameters in each situation may further increase the discriminative power of the method described in the present paper. Indeed different biological situations may impact differentially the various CF parameters, therefore the most informative CF parameters may differ among the biological situations. The selection of the most informative CF parameters in each biological situation would thus provide a first step toward specific signatures of particular stresses. This constitutes the scope of further studies in order to complete and improve the present method.

In plant phenotyping, heterogeneity linked to each plant constitutes an important and widespread limitation. In this paper, the computation method based on multiple Bhattacharyya distance calculation between tested-strain and controls on each leaf allowed to circumvent the inter-leaves heterogeneity and to take into account the heterogeneity of each phenotype. Plant-pathogen interactions studies often face phenotypes that could be hardly discriminated by visual assessment. The computation method based on the combination of images of multiple CF parameters provided an efficient discrimination of visually similar phenotypes which differed only by one protein secretion or protein expression. The results obtained in the present paper support the idea that combination of images of CF parameters improve the discrimination between distinct biotic stresses compared to single CF approach. Furthermore, the combination of several imaging techniques using this computation method could constitute an advance in the identification of specific signature of these biotic stresses.

## Data Availability Statement

The datasets generated for this study are available on request to the corresponding author.

## Author Contributions

VM, TB, and EB conceived the study, drafted the manuscript, designed the experimental protocols, and developed the computational method applied in this study. VM, LL, DS, and CB realized the experimentations. GL contributed to the management and the analysis of the chlorophyll fluorescence images. GH worked on the Bhattacharyya distance applications to data of the study. All authors read and approved the final manuscript.

### Conflict of Interest

The authors declare that the research was conducted in the absence of any commercial or financial relationships that could be construed as a potential conflict of interest.
