# Supplementary material for: A Computation Method Based on the Combination of Chlorophyll Fluorescence Parameters to Improve the Discrimination of Visually Similar Phenotypes Induced by Bacterial Virulence Factors
Source: Front Plant Sci. 2020 Feb 26;11:213. doi: 10.3389/fpls.2020.00213 (PMC7055487; doi:10.3389/fpls.2020.00213)
Supplement: Supplementary file 1 [file Data_Sheet_1.PDF]

Supplementary Material

Table 1: Bacterial strains used in this study.

name in the text	full designation	accession	genotype or relevant phenotype	reference
7698R	7698R	CFBP 13420	spontaneous ^{Rifr} derivative of strain CFBP 7698	Meline 2019
7698R pIJ3225	7698R pIJ3225	CFBP 13431	13431 pIJ3225	Meline 2019
C3	7698R pIJ3225 C3	CFBP 13465	$Rifr, Tetr, Kanr$	Meline 2019
F2	7698R pIJ3225 F2	CFBP 13468	$Rifr, Tetr, Kanr$	Meline 2019
F15	7698R pIJ3225 F15	CFBP 13467	$Rifr, Tetr, Kanr$	Meline 2019
G1	7698R pIJ3225 G1	CFBP 13470	$Rifr, Tetr, Kanr$	Meline 2019
G2	7698R pIJ3225 G2	CFBP 13472	$Rifr, Tetr, Kanr$	Meline 2019
G9	7698R pIJ3225 G9	CFBP 13474	$Rifr, Tetr, Kanr$	Meline 2019
EHA105	*Agrobacterium* EHA105 no protein express	/	$genta$	this study
GUS	*Agrobacterium* EHA105 pB7FWG2-*gus*	/	$genta, spec$	this study
AF	*Agrobacterium* EHA105 pB7FWG2-*xopAF*	/	$genta, spec$	this study
L	*Agrobacterium* EHA105 pB7FWG2-*xopL*	/	$genta, spec$	this study
G	*Agrobacterium* EHA105 pB7FWG2-*xopG*	/	$genta, spec$	this study
V	*Agrobacterium* EHA105 pB7FWG2-*xopV*	/	$genta, spec$	this study
T	*Agrobacterium* EHA105 pB7FWG2-*xopT*	/	$genta, spec$	this study
AK	*Agrobacterium* EHA105 pB7FWG2-*xopAK*	/	$genta, spec$	this study

For more information on the localisation of the various Tn5 insertions in the *hrp* cluster, please see reference Meline et al. 2019, Molecular Plant Pathology.

Table 2: PCR primers used in this study. These PCR primers were designed on *X. fuscans* pv. *citri* 4834, dedicated to this study.

targeted gene	forward primers	reverse primers	amplicon (bp)
xopAF	CACCATGGGCCTATGCATTAC	GGCGGCAACCAAATGCTT	843
xopG	CACCATGCCAATCAGTCAAACAAAC	CATGCCGTGAGGCTTATATTTTTTGCG	642
xopL	CACCATGAACGAGGCGGCTGG	CTGCTGGCCTGAAGCTTCCGG	1716
xopT	CACCATGCGCCCTCTTTCGCCC	CGCTCCAGGGTGGTTCAACC	948
xopAK	CACCATGGGTGGGACTGTTGACC	CCACGACTTGTAGTAGAAGATGC	878
xopV	CACCATGAAAATCTCCGGCTCGG	TTCGCCGTTCGGATCAGAATG	996

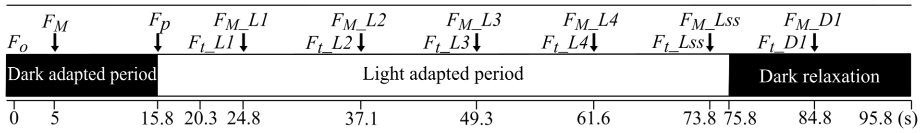

Symbol	*Formula*	*Name*
F_o	Measure	Minimal chlorophyll fluorescence intensity measured during the dark adapted state
F_o_Ln	F_o / ((F_M - F_o) / (F_M + F_o / F_M_Ln))	Minimal chlorophyll fluorescence intensity measured during the light adapted state
F_o_Lss	Fo / ((F_M - F_o) / (F_M + F_o / F_M_Lss))	Minimal chlorophyll fluorescence intensity measured during the light steady state
F_o_Dn	Measure	Minimal chlorophyll fluorescence intensity measured during the dark relaxation state
F_M	Measure	Maximal chlorophyll fluorescence intensity measured during the dark adapted state
F_M_Ln	Measure	Maximal chlorophyll fluorescence intensity measured during the light adapted state
F_M_Lss	Measure	Maximal chlorophyll fluorescence intensity measured during the light steady state
F_M_Dn	Measure	Maximal chlorophyll fluorescence intensity measured during the dark relaxation state
F_p	Measure	Peak fluoresence measured during the initial phase of the Kautsky effect
Ft_Ln	Measure	Instantaneous fluoresence intensity measured during the light adapted state
Ft_Lss	Measure	Instantaneous fluoresence intensity measured during the light steady state
Ft_Dn	Measure	Instantaneous fluoresence intensity measured during the dark relaxation state
F_v	FM - F_o	Variable fluorescence calculated during the dark adapted state
F_v_Ln	F_M - F_o_Ln	Variable fluorescence calculated during the light adapted state
F_v_Lss	F_M - F_o_Lss	Variable fluorescence calculated during the light steady state
F_q_Ln	F_M_Ln - F_t_Ln	Variable fluorescence calculated during the light adapted state
F_q_Lss	F_M_Lss - F_t_Lss	Variable fluorescence calculated during the light steady state
NPQ _Ln	(F_M - F_M_Ln) / (F_M_Ln)	Non photochemical quenching calculated during the light adapted state
NPQ _Lss	(F_M - F_M_Lss) / (F_M_Lss)	Non photochemical quenching calculated during the light steady state
NPQ _Dn	(F_M - F_M_Dn) / (F_M_Dn)	Non photochemical quenching calculated during the dark relaxation state
Qy_Ln	(F_M_Ln - F_t_Ln) / F_M_Ln	Instantaneous PSII quantum yield calculated during the light adapted state
Qy_Lss	(F_M_Lss - F_t_Lss) / F_M_Lss	Instantaneous PSII quantum yield calculated during the light steady state
Qy_Dn	(F_M_Dn - F_t_Dn) / F_M_Dn	Instantaneous PSII quantum yield calculated during the dark relaxation state
F_vF_M	(F_M - F_o) / F_M	PSII quantum yield calculated during the dark adapted state
F_vF_M_Ln	(F_M_Ln - F_o_Lss) / F_M_Ln	PSII quantum yield calculated during the light adapted state
F_vF_M_Lss	(F_M_Lss - F_o_Lss) / F_M_Lss	PSII quantum yield calculated during the light steady state
qP_Ln	(F_M_Ln - F_t_Ln) / (F_M_Ln - F_o_Ln)	PSII Coefficient of photochemical quenching calculated during the light adapted state
qP_Lss	(F_M_Lss - F_t_Lss) / (F_M_Lss - F_o_Lss)	PSII Coefficient of photochemical quenching calculated during the light steady state
qP_Dn	(F_M_Dn - F_t_Dn) / (F_M_Dn - F_o_Dn)	PSII Coefficient of photochemical quenching calculated during the dark relaxation state
qP_Ln	(F_M_Ln - F_t_Ln) / (F_M_Ln - F_o_Ln)	PSII Coefficient of photochemical quenching calculated during the light adapted state
qP_Lss	(F_M_Lss - F_t_Lss) / (F_M_Lss - F_o_Lss)	PSII Coefficient of photochemical quenching calculated during the light steady state
qL_Ln	(Fq_Ln / Fv_Ln) / (Fo / Ft)	PSII Coefficient of photochemical quenching calculated during the dark relaxation state
Rfd _Ln	(F_p - F_t_Ln) / (F_t_Ln)	Fluorescence decline ratio calculated during the light adapted state
Rfd_Lss	(F_p - F_t_Lss) / (F_t_Lss)	Fluorescence decline ratio calculated during the light steady state

Figure S1. A description of the CF quenching protocol providing the measured and calculated images of CF parameters used in this study. To measure F_0, a modulated light of 0.1 μmol.m^{-2}.s^{-1} is used. Then 618 nm orange actinic light with intensities of 20% of the 400 μmol.m^{-2}.s^{-1} is used during the light-adapted period of 60 sec. The protocol also provides 6 pulses of 0.8 sec duration of 455nm blue saturating light with an intensity of 50% of the 3000 μmol.m^{-2}.s^{-1}: 5 pulses during the light-adapted period and 1 pulse during the dark-relaxation period. The whole duration of the illumination protocol is of 95.8 sec.
Twelve parameters are used in the analysis: F_0, F_m, F_p, Ft, F_v, F_q, NPQ, Qy, qP, qL, Rfd and F_v/F_m. Among these 12 parameters, F_0, F_m, F_p, Ft, F_v are measured to generate images. For each measured parameters, several measures/images are performed during the illumination process, as depicted in the upper part of the figure S1. For example, for parameter F_m, the initial measurement Fm is measured after 5 seconds during the dark-adapted period. Then, 5 measurements are realised during the light-adapted period (F_mL1 to F_mL4 + F_mLss). One last measurement is done during dark relaxation (F_mD1). The precise timing of the measurment is mentioned in the X-axis of the chart. The other parameters are calculated according to the formulas. In total, the measures performed lead to the generation of 70 images, corresponding to the various parameters used in the analysis.

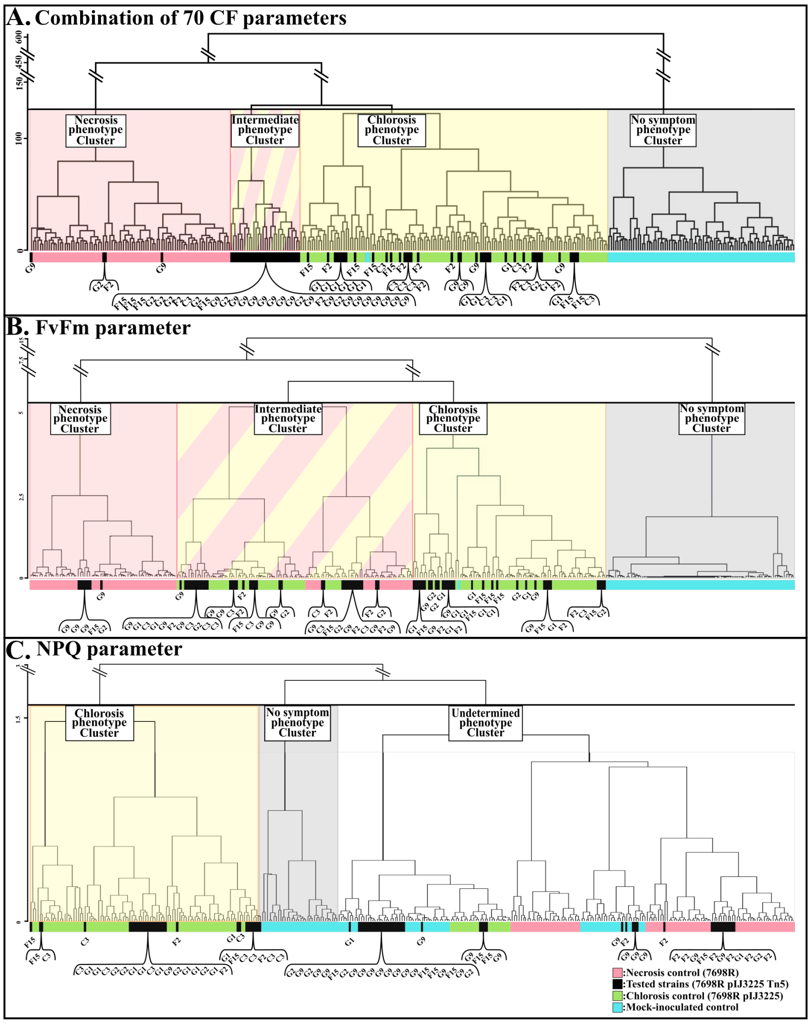

Figure S2. Dendrograms obtained from the computation method according to the use of a combination of the 70 images of CF parameters (A.), sole F_v/F_m parameter (B.) and sole NPQ parameter (C.) respectively. Dendrograms are based on three-dimensional Euclidean distances evaluation and Ward agglomeration method.
